# The role of established East Asian obesity-related loci on pediatric leptin levels highlights a neuronal influence on body weight regulation in Chinese children and adolescents: the BCAMS study

**DOI:** 10.18632/oncotarget.20547

**Published:** 2017-08-24

**Authors:** Junling Fu, Ge Li, Lujiao Li, Jinhua Yin, Hong Cheng, Lanwen Han, Qian Zhang, Naishi Li, Xinhua Xiao, Struan F.A. Grant, Mingyao Li, Shan Gao, Jie Mi, Ming Li

**Affiliations:** ^1^ Department of Endocrinology, Key Laboratory of Endocrinology, National Health and Family Planning Commission, Peking Union Medical College Hospital, Chinese Academy of Medical Sciences and Peking Union Medical College (CAMS & PUMC), Beijing, P.R. China; ^2^ Department of Epidemiology, Capital Institute of Paediatrics, Beijing, P.R. China; ^3^ Department of Endocrinology, Chaoyang Hospital, Capital Medical University, Beijing, P.R. China; ^4^ Division of Endocrinology, The Children’s Hospital of Philadelphia, Perelman School of Medicine, University of Pennsylvania, Philadelphia, PA, USA; ^5^ Division of Human Genetics, The Children’s Hospital of Philadelphia Research Institute, Philadelphia, PA, USA; ^6^ Department of Pediatrics, Perelman School of Medicine, University of Pennsylvania, Philadelphia, PA, USA; ^7^ Department of Biostatistics and Epidemiology, University of Pennsylvania, Philadelphia, PA, USA

**Keywords:** obesity, SNP, leptin, adipokine, children

## Abstract

Genome-wide association studies have identified multiple variants associated with adult obesity, mostly in European-ancestry populations. We aimed to systematically assess the contribution of key loci, which had been previously shown to be associated in East Asian adults, to childhood obesity, related adipokine profiles and metabolic traits in a Chinese pediatric population. Twelve single-nucleotide polymorphisms (SNPs) plus metabolic profiles and levels of five adipokines (leptin, adiponectin, resistin, fibroblast growth factor 21 and retinol binding protein 4) were evaluated in 3,506 Chinese children and adolescents aged 6-18. After correction for multiple comparisons, six of these SNPs were robustly associated with childhood obesity: *FTO*-rs1558902 (*P*=5.6×10^−5^), *MC4R*-rs2331841 (*P*=4.4×10^−4^), *GNPDA2*-rs16858082 (*P* = 3.4×10^−4^), *PCSK1*-rs261967 (*P* = 0.001), *SEC16B*-rs516636 (*P* = 0.004) and *MAP2K5*-rs4776970 (*P* = 0.004), with odds ratios ranging from 1.211 to 1.421; while *ITIH4*-rs2535633 and *BDNF*-rs2030323 yielded nominal association with the same trait (*P* < 0.05). Moreover, the risk alleles of six SNPs displayed significant (*P* < 0.004) or nominal (*P* < 0.05) association with leptin levels, namely at in/near *PCSK1, MC4R, FTO, MAP2K5, GNPDA2* and *BDNF* plus their cumulative genetic score yielded stronger association with increased leptin levels (*P* = 6.2×10^−11^). Our results reveal that key obesity-associated loci previously reported in Europeans, but also associated with East Asian adults, are also associated with obesity and/or metabolic quantitative traits in Chinese children. These associations coincide with six brain-expressed loci that correlate with leptin levels, thus may point to an important neuronal influence on body weight regulation in the pediatric setting.

## INTRODUCTION

The worldwide epidemic of obesity poses a major risk for the development of type 2 diabetes (T2D), cardiovascular disease (CVD), hypertension, stroke and certain types of cancer [1, 2]. In China, obesity rates have increased sharply over the past 20-30 years, particularly in childhood, rapidly climbing from 0.2% in 1985 to 8.1% in 2012 [3]. Both genetic predisposition and lifestyle factors are known to contribute to the risk of obesity [4]. To date, genome-wide association studies (GWAS) have identified numerical loci that contribute to body weight in adults and children of diverse ancestry [5-16]. However, the majority of these studies were conducted in populations of European ancestry whereas studies of pediatric obesity in Asians remain limited [11-14]. Moreover, recent reports of obesity-related loci enriched in brain-expressed genes such as *FTO, MC4R*, *GNPDA2,* and *BDNF* point to a neuronal influence on body weight regulation in adults [17]. We queried whether such an influence existed, or was even more pronounced in children. Therefore, we selected thirteen of the strongest BMI-related loci reported from a recent meta-analysis of GWAS in adult of East Asian-ancestry populations [12-14] to test their associations with obesity in a well-established cohort of Chinese school-aged children, with particularly focus on the single-nucleotide polymorphisms (SNPs) related to brain-expressed loci.

In parallel, recent studies have shown that obesity-related cadiometabolic risk can be mediated by the pleiotropic effects of adipokines, the secretory products that reflect endocrine function in adipose tissue [18], particularly by these adipokines which may function via the central nervous system (CNS), such as leptin, adiponectin, resistin, fibroblast growth factor 21 (FGF21) [19] and retinol binding protein 4 (RBP4) [20]. We therefore elected to investigate if these selected genetic variants are associates with a set of adipokines, as well as other cardiometabolic traits, in an attempt to gain novel insight in to their role in both obesity and cardiometabolic dysregulation.

## RESULTS

### Cohort characteristics

General characteristics of the study subjects are listed in Table 1. The study was conducted in 3,506 including normal weight (n = 1,626), overweight (n = 654), and obese (n = 1,226) unrelated individuals recruited through the BCAMS study. 51% of the participants were male, and the mean age was 12.4 ± 3.1 years. Obesity-related traits, blood pressures, triglyceride (TG), low density lipoprotein cholesterol (LDL-C), fasting insulin and homeostasis model assessment of insulin resistance (HOMA-IR) increased, while high density lipoprotein cholesterol (HDL-C) decreased, with increasing body mass index (BMI) (all *P* < 0.001). As expected, obese children exhibited highest leptin, resistin and RBP4 levels, and lowest adiponectin and FGF21 concentrations compared with the other two groups (all *P* < 0.001).

**Table 1:** Descriptive statistics of phenotypes used in the study

Phenotype	Mean (SD, %)				*P*
	All	Normal weight	Overweight	Obese	
Male/n (%)	1787/3506 (51%)	674/1626 (41.5%)	328/654 (50.2%)	785/1226(64%)	**< 0.001**
**Anthropometric parameters**					
Age (yrs)	12.4 ± 3.1	12.45 ± 3.16	13.29 ± 2.97 ^*^	11.80 ± 2.88 ^*^+	**< 0.001**
Puberty stages	2.68 ± 1.43	2.68 ± 1.36	3.16 ± 1.44 ^*^	2.41 ± 1.44 ^*^+	**< 0.001**
BMI (kg/m^2^)	21.9 ± 4.9	17.78 ± 2.43	23.41 ± 2.48^*^	26.57 ± 3.66 ^*^+	**< 0.001**
WC (cm)	72.4 ± 13.1	62.24 ± 7.14	76.20 ± 7.79 ^*^	83.73 ± 10.84 ^*^+	**< 0.001**
Percent body fat	24. 4 ± 8.54	18.0 ± 5.64	27.8 ± 6.12^*^	30.8 ± 6.66^*^+	**< 0.001**
UAC (cm)	25.4 ± 4.94	21.7 ± 3.23	27.2 ± 3.05^*^	29.3 ± 3.95^*^+	**< 0.001**
SBP (mmHg)	108 ± 14	101± 12	111± 12^*^	114± 13 ^*^+	**< 0.001**
DBP (mmHg)	68 ± 10	64± 10	69± 9 ^*^	72 ± 9 ^*^+	**< 0.001**
**Biochemical parameters**					
TC (mmol/L)	4.09 ± 0.82	4.12 ± 0.90	4.03 ± 0.76 ^*^	4.09 ± 0.74 ^*^	**0.040**
TG (mmol/L)	0.93 (0.91-0.94)	0.81 (0.80-0.83)	0.95 (0.92-0.99) ^*^	1.08 (1.05-1.10) ^*^+	**< 0.001**
LDL-C (mmol/L)	2.55 ± 0.75	2.49 ± 0.84	2.53 ± 0.69	2.63 ± 0.66 ^*^+	**< 0.001**
HDL-C (mmol/L)	1.40 ± 0.32	1.53 ± 0.32	1.33 ± 0.29 ^*^	1.27 ± 0.26 ^*^+	**< 0.001**
FBG (mmol/L)	5.08 ± 0.55	5.00 ± 0.47	5.16 ± 0.79 ^*^	5.15 ± 0.48 ^*^	**< 0.001**
Fasting insulin (mU/L)	8.19 (7.97-8.41)	5.63 (5.43-5.83)	9.43 (8.99-9.89) ^*^	12.05 (11.61-12.54) ^*^+	**< 0.001**
HOMA-IR	1.84 (1.80-1.90)	1.25 (1.20-1.30)	2.15 (2.05-2.26) ^*^	2.75 (2.64-2.87) ^*^+	**< 0.001**
Adipokines					
Leptin (ng /ml)	5.18 (4.93-5.41)	2.05 (1.93-2.18)	7.76 (7.26-8.40) ^*^	13.17 (12.59-13.77) ^*^+	**< 0.001**
Adiponectin (ug/ml)	5.41 (5.30-5.52)	6.46 (6.28-6.66)	4.89 (4.68-5.12) ^*^	4.57 (4.42-4.71) ^*^+	**< 0.001**
Resistin (ng /ml)	15.36 (15.06-15.63)	14.82 (14.40-15.26)	15.43 (14.75-16.08)	16.01 (15.50-16.52) ^*^	**< 0.001**
FGF21 (pg/ml)	619.8 (594.0-650.1)	676.9 (635.0-730.4)	628.3 (568.4-692.7)	552.0 (513.0-595.9) ^*^	**< 0.001**
RBP4 (ug/ml)	32.1 (31.7-32.5)	29.75 (29.19-30.31)	33.6 (32.7-34.5) ^*^	34.5(33.8-35.1) ^*^	**< 0.001**

Data were expressed as mean ± SD, n (%). Skewed distributions were expressed as geometric mean (95% confidence interval). One-way ANOVA where differences versus normal weight subjects are indicated as ^*^P < 0.05, differences versus overweight individuals are indicated as + *P* < 0.05.

Abbreviation: BMI, body mass index; WC, waist circumference; UAC, upper arm circumference; SBP, systolic blood pressure; DBP, diastolic blood pressure; TC, total cholesterol; TG, triglycerides; LDL-C, low density lipoprotein cholesterol; HDL-C, high-density lipoprotein cholesterol; FBG, fasting blood glucose; HOMA-IR, homeostasis model assessment for insulin resistance; FGF21, fibroblast growth factor 21 and RBP4, retinol binding protein 4.

### Replication with childhood obesity

The allele frequencies of the SNPs and the results of genotypic tests are listed in Table 2. The minor allele frequencies of the twelve SNPs utilized ranged from 10% to 47%. Using the additive model, six SNPs revealed significant associations with obesity after multiple testing correction (Bonferroni corrected *P* < 0.004), with odds ratios (ORs) from 1.211 to 1.421, namely *FTO*-rs1558902 (*P* = 5.6×10^−5^), *MC4R*-rs2331841 (*P* = 4.4×10^−4^), *GNPDA2*-rs16858082(*P* = 3.4×10^−4^), *PCSK1-*rs261967 (*P* = 0.001), *SEC16B*-rs516636 (*P* = 0.004), and *MAP2K5*-rs4776970 (*P* = 0.004), while other two loci, *ITIH4-*rs2535633 (*P* = 0.012) and *BDNF-*rs2030323(*P* = 0.031), showed nominal association with obesity, with ORs 1.157 and 1.133, respectively. Similar trends were also evident when combining overweight and obesity status and comparing them with the normal weight group (normal weight vs. overweight & obesity).

**Table 2:** Associations of SNPs with childhood obesity

SNP	Nearest gene		a/A^*^	MAF^†^	P (HWE)^‡^	Normal vs Obesity		Normal vs Overweight & Obesity		BMI (kg/m^2^)	
						OR(95% CI)	*P* ^§^	OR (95% CI)	*P* ^§^	β (95% CI)	*P* ^§^
rs1558902	*FTO*	intron	T/A	0.10	0.02	1.421 (1.198-1.686)	**5.6E-5**	1.405 (1.204-1.639)	**1.58E-5**	0.831 (0.509-1.154)	**4.6E-7**
rs2331841	*MC4R*	near	A/G	0.22	0.82	1.267 (1.111-1.445)	**4.4E-4**	1.212 (1.079-1.363)	**0.001**	0.486 (0.238-0.735)	**1.3E-4**
rs16858082	*GNPDA2*	near	C/T	0.34	0.56	1.239 (1.102-1.393)	**3.4E-4**	1.220 (1.100-1.353)	**1.68E-4**	0.384 (0.164-0.604)	**0.001**
rs261967	*PCSK1*	intron	A/C	0.41	0.24	1.212 (1.081-1.359)	**0.001**	1.164 (1.052-1.288)	**0.003**	0.311 (0.095-0.528)	**0.005**
rs516636	*SEC16B*	near	C/A	0.20	0.38	1.221 (1.064-1.401)	**0.004**	1.224 (1.082-1.384)	**0.001**	0.371 (0.110-0.631)	**0.005**
rs4776970	*MAP2K5*	intron	T/A	0.22	0.01	1.211 (1.063-1.379)	**0.004**	1.207 (1.076-1.355)	**0.001**	0.347 (0.101-0.594)	**0.006**
rs2535633	*ITIH4*	intron	C/G	0.41	0.52	1.157 (1.033-1.295)	**0.012**	1.122 (1.015-1.241)	**0.024**	0.221 (0.006-0.436)	**0.044**
rs2030323	*BDNF*	intron	T/G	0.47	0.95	1.133 (1.011-1.270)	**0.031**	1.117 (1.011-1.235)	**0.030**	0.216 (0.004-0.428)	**0.046**
rs6545814	*ADCY3/RBJ*	intron	A/G	0.41	0.89	1.107 (0.989-1.240)	0.078	1.073 (0.969-1.187)	0.175	0.219 (0.002-0.435)	**0.047**
rs652722	*PAX6*	intron	T/C	0.34	0.75	0.937 (0.832-1.056)	0.287	0.936 (0.843-1.040)	0.219	-0.118 (-0.343-0.107)	0.303
rs12597579	*GP2*	intron	T/C	0.28	0.52	1.069 (0.942-1.213)	0.303	1.049 (0.939-1.173)	0.395	0.139 (-0.099-0.378)	0.252
rs2237892	*KCNQ1*	intron	T/C	0.31	0.07	0.983 (0.872-1.109)	0.783	0.982 (0.883-1.092)	0.737	0.052 (-0.176-0.279)	0.655
12 loci	*GPS*_*all*_	/	/	/	/	1.150 (1.108-1.194)	**2.4E-13**	1.128 (1.091-1.166)	**9.1E-13**	0.261 (0.193-0.330)	**9.4E-14**

^*^a: non-effect; A: effect; † minor allele frequency; ‡*P* value for Hardy-Weinberg equilibrium test; OR/β (95% CI) and §P value for logistic/linear regression in the additive model were adjusted for gender, age, Tanner stage and residence.

Abbreviation: *MC4R*, melanocortin 4 receptor; *FTO*, fat mass and obesity associated; *GNPDA2*, glucosamine-6-phosphate deaminase 2; *PCSK1*, proprotein convertase subtilisin/kexin type 1; *SEC16B*, SEC16 homolog B, endoplasmic reticulum export factor; *MAP2K5*, mitogen-activated protein kinase 5; *ITIH4*, inter-alpha-trypsin inhibitor heavy chain family, member 4; *BDNF*, brain-derived neurotrophic factor; *ADCY3/RBJ,* adenylate cyclase 3; *PAX6*, paired box 6; *GP2*, glycoprotein 2; *KCNQ1*, potassium channel, voltage gated KQT-like subfamily Q, member 1 and GPS_all_, sum gene score of all 12 loci.

Note: Values in bold are significant at *P* < 0.05

Similarly, we found strong associations between BMI and variants in/near *FTO* (*P =* 4.6×10^−7^), *MC4R* (*P =* 1.3×10^−4^), and *GNPDA2* (*P =* 0.001), and nominal association in/near *SEC16B, PCSK1*, *MAP2K5*, *ITIH4*, *BDNF* and *ADCY3/RBJ* (*P* < 0.05), with the effects ranging from 0.83 kg/m^2^ to 0.22 kg/m^2^ per allele (Table 2). Moreover, we found similar effects of BMI-related loci on other obesity-related anthropometric traits i.e. waist circumference (WC), percent body fat and upper arm circumference (UAC) (Table 5), which was largely consistent with the above observations with obesity risk. The genetic predisposition score consisting of all twelve adult BMI-associated variants (GPS_all_) was more significantly associated with BMI (*P* = 9.40×10^−14^), WC (P = 1.42×10^−14^), percent body fat (6.10×10^−11^) and UAC (3.50×10^−12^) than with any single SNP.

**Table 5:** Associations of the 12 SNPs and GPSs with obesity-related and other cardio-metabolic traits

Gene	SNP		WC (cm)	Percent body fat	UAC (cm)	SBP (mmHg)	DBP (mmHg)	TC (mmol/L)	TG (mmol/L)^#^	LDL-C (mmol/L)	HDL-C (mmol/L)	FBG (mmol/L)	Insulin (mU/L)^#^	HOMA-IR^#^
*FTO*	rs1558902	*β*	1.887	1.150	0.857	1.491	0.964	-0.020	0.007	-0.007	-0.011	-0.011	0.058	0.057
		*P*	**6.3E-6**	**1.5E-4**	**2.5E-8**	**0.001**	0.005	0.496	0.683	0.807	0.322	0.599	0.031	0.044
*MC4R*	rs2331841	*β*	1.183	0.847	0.482	0.588	0.300	-0.007	0.026	-0.001	-0.012	0.046	0.064	0.072
		*P*	**2.3E-4**	**2.8E-4**	**4.7E-5**	0.096	0.260	0.757	0.045	0.946	0.184	**0.004**	**0.002**	**0.001**
*GNPDA2*	rs16858082	*β*	0.974	0.665	0.265	0.852	0.421	-0.048	0.005	-0.030	-0.021	0.017	0.056	0.059
		*P*	**0.001**	**0.001**	0.011	0.006	0.074	0.017	0.643	0.109	0.006	0.231	**0.002**	**0.002**
*SEC16B*	rs516636	*β*	0.972	0.493	0.279	0.575	0.148	0.003	0.015	0.008	-0.012	0.011	0.039	0.041
		*P*	**0.004**	0.044	0.025	0.121	0.595	0.884	0.268	0.715	0.180	0.518	0.073	0.068
*PCSK1*	rs261967	*β*	0.790	0.339	0.287	0.406	0.508	-0.042	0.009	-0.024	-0.015	-0.014	0.042	0.041
		*P*	0.005	0.091	0.005	0.186	0.028	0.031	0.414	0.189	0.049	0.292	0.020	0.028
*MAP2K5*	rs4776970	*β*	0.982	0.704	0.338	0.709	0.424	4.5E-4	0.030	0.001	-0.023	-0.015	0.035	0.032
		*P*	**0.002**	**0.002**	**0.004**	0.043	0.107	0.984	0.017	0.978	0.010	0.321	0.086	0.140
*ITIH4*	rs2535633	*β*	0.586	0.430	0.165	0.266	0.109	0.033	0.019	0.037	-0.006	-0.001	0.036	0.035
		*P*	0.035	0.033	0.107	0.384	0.634	0.092	0.084	0.032	0.396	0.939	0.045	0.060
*BDNF*	rs2030323	*β*	0.386	0.343	0.191	-0.284	-0.262	0.016	0.006	0.018	-0.004	-0.004	0.029	0.027
		*P*	0.163	0.087	0.062	0.351	0.254	0.418	0.615	0.326	0.600	0.763	0,105	0.141
*ADCY3* *RBJ*	rs6545814	*β*	0.599	0.367	0.177	0.385	0.395	-0.007	0.002	0.009	-0.008	-0.019	0.025	0.023
		*P*	0.032	0.070	0.086	0.211	0.088	0.728	0.865	0.625	0.290	0.160	0.157	0.226
*KCNQ1*	rs2237892	*β*	0.271	0.135	0.048	-0.231	0.038	-0.041	0.020	-0.027	-0.028	0.016	0.008	0.011
		*P*	0.356	0.527	0.659	0.475	0.876	0.046	0.094	0.159	**0.001**	0.257	0.687	0.587
*PAX6*	rs652722	*β*	-0.143	-0.044	-0.132	0.127	0.053	-0.016	-0.014	-0.010	-0.004	-0.017	-0.010	-0.013
		*P*	0.622	0.834	0.218	0.690	0.825	0.439	0.223	0.584	0.647	0.225	0.601	0.511
*GP2*	rs12597579	*β*	0.474	0.164	0.119	0.168	0.183	0.016	0.010	0.009	-3.5E-4	0.002	-0.004	-0.003
		*P*	0.124	0.462	0.294	0.619	0.472	0.471	0.396	0.662	0.967	0.921	0.857	0.869
GPS_all_	all 12 loci	*β*	0.683	0.418	0.226	0.397	0.26	-0.009	0.011	1.2E-4	-0.013	0.001	0.033	0.033
		*P*	**1.4E-14**	**6.1E-11**	**3.5E-12**	**4.7E-5**	**4.1E-4**	0.178	**0.002**	0.983	**2.7E-7**	0.735	**7.3E-9**	**1.7E-8**
		*EV*	1.3%	1.2%	1.0%	0.4%	0.3%	< 0.01%	0.3%	< 0.01%	0.8%	< 0.01%	0.9%	0.8%
GPS_leptin_	six leptin-related loci	*β*	0.972	0.608	0.352	0.523	0.315	-0.017	0.013	-0.007	-0.015	0.004	0.046	0.047
		*P*	**2.3E-13**	**3.4E-11**	**4.1E-14**	**1.8E-4**	**0.003**	0.050	0.008	0.392	**2.3E-5**	0.529	**1.2E-8**	**2.9E-8**
		*EV*	1.3%	1.2%	1.2%	0.4%	0.3%	< 0.01%	0.2%	< 0.01%	0.6%	< 0.01%	1.0%	1.0%

β means linear regression coefficients adjusted for gender, age, Tanner stage and residence.

Abbreviation: BMI, body mass index; WC, waist circumference; UAC, upper arm circumference; SBP, systolic blood pressure; DBP, diastolic blood pressure; TC, total cholesterol; TG, triglycerides; LDL-C, low-density lipoprotein cholesterol; HDL-C, high-density lipoprotein cholesterol; FBG, fasting blood glucose; HOMA-IR, homeostasis model assessment for insulin resistance; *EV,* explained variation; *FTO*, fat mass and obesity associated; *MC4R*, melanocortin 4 receptor; *GNPDA2*, glucosamine-6-phosphate deaminase 2; *SEC16B,* SEC16 homolog B, endoplasmic reticulum export factor; *PCSK1*, proprotein convertase subtilisin/kexin type 1; *MAP2K5*, mitogen-activated protein kinase 5; *ITIH4,* inter-alpha-trypsin inhibitor heavy chain family, member 4; *BDNF*, brain-derived neurotrophic factor; *ADCY3/RBJ*, adenylate cyclase 3; *KCNQ1*, potassium channel, voltage gated KQT-like subfamily Q, member 1; *PAX6*, paired box 6; *GP2,* glycoprotein 2; GPS_all_, sum gene score of all 12 loci and GPS_leptin_, sum gene score of six leptin-related loci.

^#^ Skewed distributions were natural logarithmically (ln) transformed.

Note: Values in bold are significant at *P* < 0.004 after Bonferroni correction.

### Biological mechanisms for obesity predisposing genes

In order to examine biological mechanisms for obesity predisposing genes, we selected the SNPs associated with BMI/obesity (at least one of these traits) from above analysis, plus all the 12 SNP-based gene score (GPS_all_) and tested their association with the 5 adipokines. As shown in Table 3, we found the risk alleles of *FTO*-rs1558902 (*P* = 0.002), *MAP2K5-*rs4776970 (*P* = 0.002) and *MC4R-*rs2331841 (*P* = 0.003) yielded significant association with increasing leptin levels at a Bonferroni-corrected threshold of *P* ≤ 0.004, while the risk alleles of *GNPDA2-*rs16858082 (*P* = 0.007), *PCSK1-*rs261967 (*P* = 0.007) and *BDNF-*rs2030323 (*P* = 0.027) yielded nominal association with leptin levels. In addition, the risk alleles of *MC4R-*rs2331841 (*P* = 0.020) and *BDNF-*rs2030323 (*P* = 0.032) displayed nominal association with decreased adiponectin levels, while *PCSK1-*rs261967 showed a nominal association with increased adiponectin levels (*P* = 0.038). The GPS_all_ was more significantly associated with leptin levels (*P* = 9.70×10^−10^). However, neither GPS_all_ nor any individual single SNP was associated with the levels of other adipokines.

**Table 3:** Associations of the selected SNPs and GPS with five adipokines

Gene	SNP		Leptin (ng/ml)^#^	Adiponectin (ug/ml)^#^	Resistin (ng/ml)^#^	FGF21 (pg/ml)^#^	RBP4 (ug/ml)^#^
*FTO*	rs1558902	*β*	0.149	-0.027	0.019	0.022	0.004
		*P*	**0.002**	0.202	0.342	0.664	0.760
*MC4R*	rs2331841	*β*	0.110	-0.038	0.011	0.012	0.009
		*P*	**0.003**	0.020	0.454	0.764	0.340
*GNPDA2*	rs16858082	*β*	0.089	-0.004	-0.005	-0.006	0.003
		*P*	0.007	0.800	0.721	0.815	0.736
*SEC16B*	rs516636	*β*	0.067	-0.025	0.013	-0.059	0.015
		*P*	0.088	0.153	0.402	0.150	0.157
*PCSK1*	rs261967	*β*	0.087	0.030	0.012	-0.005	0.007
		*P*	0.007	0.038	0.350	0.882	0.423
MAP2K5	rs4776970	β	0.114	-0.020	0.006	-0.012	0.014
		P	**0.002**	0.212	0.674	0.749	0.138
*ITIH4*	rs2535633	*β*	0.050	-0.005	-0.022	-0.006	0.011
		*P*	0.118	0.719	0.094	0.868	0.185
*BDNF*	rs2030323	*β*	0.071	-0.030	0.005	-0.035	-0.007
		*P*	0.027	0.032	0.683	0.291	0.414
*ADCY3/RBJ*	rs6545814	*β*	0.050	-0.016	-0.013	-0.024	3.79E-4
		*P*	0.123	0.277	0.323	0.469	0.966
*GPS*_*all*_	all 12 loci	*β*	0.062	-0.010	0.001	-0.02	0.004
		*P*	**9.7E-10**	0.022	0.807	0.064	0.128
		*Explained variation*	1.0%	0.1%	< 0.01%	< 0.01%	< 0.01%

β means linear regression coefficients adjusted for gender, age, Tanner stage and residence.

Abbreviation: FGF21, fibroblast growth factor 21; RBP4, retinol binding protein 4; *FTO*, fat mass and obesity associated; *MC4R*, melanocortin 4 receptor; *GNPDA2*, glucosamine-6-phosphate deaminase 2; *SEC16B,* SEC16 homolog B, endoplasmic reticulum export factor; *PCSK1*, proprotein convertase subtilisin/kexin type 1; *MAP2K5*, mitogen-activated protein kinase 5; *ITIH4,* inter-alpha-trypsin inhibitor heavy chain family, member 4; *BDNF*, brain-derived neurotrophic factor ; *ADCY3/RBJ,* adenylate cyclase 3 and GPS_all_, sum gene score of all 12 loci.

^#^ Skewed distributions were natural logarithmically (ln) transformed.

Note: Values in bold are significant at *P* < 0.004 after Bonferroni correction.

Next, we selected all the variants at the leptin-associated loci for further analysis. In the General Linear Model analyses (Table 4), after adjusting for age, sex, Tanner stage and residence, leptin levels were significantly increased in subjects with the minor alleles of *PCSK1-*rs261067*, MC4R-*rs2331841, *FTO-*rs1558902, *MAP2K5-*rs4776970 and *GNPDA2-*rs16858082, respectively (0.003 ≤ *P* ≤ 0.019), while there was a trend for an increase in subjects with the minor allele of *BDNF-*rs2030323 (*P* = 0.053), with effect sizes ranging from 0.2% to 0.4%. The GPS consisted of the six leptin-associated SNPs (GPS_leptin_) showed much stronger association with increased leptin levels (*P* = 2.8×10^−6^), with an increased effect size of 1.6%. Meanwhile, leptin levels in the top tertiles of GPS_leptin_ (with 5-9 risk alleles) were increased by 31% compared to those in the lowest tertiles (with less than 3 risk alleles) (*P* < 0.001). In addition, GPS_leptin_ was able to explain an estimated 1.5%, 1.3%, 1.2% and 1.2% of the total variance of BMI, WC, percent body fat, and UAC, respectively, thus the variations explained by GPS_leptin_, reaching or exceeding the cumulated effects of all twelve loci (i.e. GPS _all)_, explaining 1.4 %, 1.3%, 1.2%, and 1.0% of the total variance, respectively (Table 5).

**Table 4:** Associations of selected loci with leptin levels

*Gene*	Gene expression abundance in hypothalamus^*^	SNP	Effect size	Leptin (ng/ml) [geometric mean (95% CI)]			*P*_*adjusted1*_	*P*_*adjusted1+*BMI_
				*aa*	*aA*	*AA*		
*PCSK1*	32.4 (10.3)	rs261967	0.4%	4.6 (4.2-5.0)	5.3 (5.0-5.7)	5.2 (4.7-5.8)	**0.003**	0.419
*MC4R*	3.8 (3.45)	rs2331841	0.3%	4.7 (4.5-5.0)	5.5 (5.1-5.8)	5.6 (4.7-6.6)	**0.004**	0.694
*FTO*	543.45 (119.7)	rs1558902	0.3%	4.9 (4.6-5.1)	5.7 (5.2-6.3)	6.0 (4.1-8.7)	**0.008**	0.216
*MAP2K5*	6.0 (5.6)	rs4776970	0.3%	4.8 (4.5-5.1)	5.4 (5.0-5.8)	6.0(5.2-7.0)	**0.013**	0.151
*GNPDA2*	6.6 (6.65)	rs16858082	0.2%	4.7 (4.4-5.1)	5.2 (4.9-5.6)	5.4 (4.8-6.2)	**0.019**	0.838
*BDNF*	12.4 (5.9)	rs2030323	0.2%	4.8 (4.3-5.3)	4.9 (4.6-5.2)	5.5 (5.0-5.9)	0.053	0.235
GPS_leptin_	/	six leptin-related loci	1.6%	Tertile1 (0-3 risk alleles) (n = 1373)	Tertile2 (4 risk alleles) (n = 828)	Tertile3 (5-9 risk alleles) (n = 1130)	**2.8E-6**	0.218
				4.4 (4.1-4.8)	5.1 (4.7-5.6)	5.8 (5.4-6.3)		

Abbreviation: *PCSK1*, proprotein convertase subtilisin/kexin type 1; *MC4R*, melanocortin 4 receptor; *FTO*, fat mass and obesity associated; *MAP2K5*, mitogen-activated protein kinase 5; *GNPDA2*, glucosamine-6-phosphate deaminase 2; *BDNF*, brain-derived neurotrophic factor and GPS_leptin_, sum gene score of six leptin-related loci.

^*^Data were expressed as mean (median in all tissues and cells), from http://biogps.org.

*P*_*adjusted1*_ means the analysis of covariance in the general linear model after adjusting for gender, age, Tanner stage and residence.

*P*_*adjusted1+*BMI_: further adjusted for BMI.

Note: Values in bold are significant at *P* < 0.05.

Interestingly, as listed in Table 4, all the genes at these leptin-increasing loci were found to be expressed in CNS.

### Best fitting model for the association between SNPs/gene score, leptin level and BMI

Since leptin was the only adipokine revealing Bonferroni-significant association with the SNPs, we therefore focused on the associations between leptin levels, leptin-related SNPs/GPS_leptin_ and BMI to test which triangular association model fitted the best: mediation, pleiotropy or moderation.

**Figure 1:**
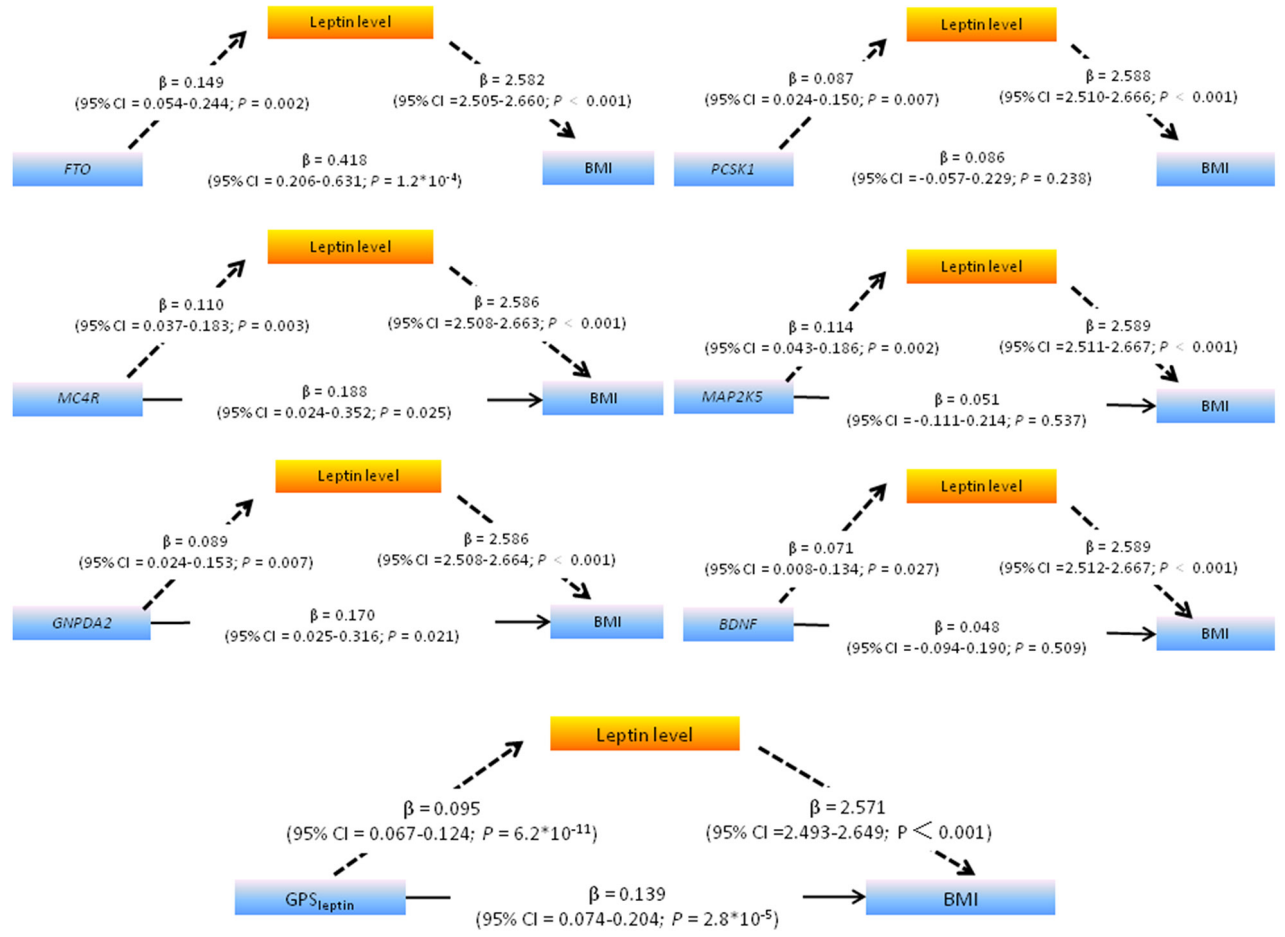
Path diagram showing that leptin significantly mediates the association between leptin-related SNPs /GPS_leptin_ (including six leptin-related SNPs) and BMI The path diagram shows the simple association between the six leptin-related loci *FTO, MC4R, GNPDA2 , PCSK1, MAP2K5, BDNF* and GPS_leptin_ and ln-leptin (β, 0.149, 0.110, 0.089, 0.087, 0.114, 0.071,and 0.095 respectively), the association between the *FTO, MC4R, GNPDA2 , PCSK1, MAP2K5, BDNF* and GPS_leptin_ and BMI adjusted for leptin (β, 0.418, 0.188, 0.170, 0.086, 0.051, 0.048, and 0.139, respectively), and the association between leptin and BMI adjusted for the individual SNP or GPS_leptin_ (β, 2.582, 2.586, 2.586, 2.588, 2.589, 2.589 and 2.571, respectively). The simple association between the *FTO, MC4R, GNPDA2, PCSK1, MAP2K5,* GPS_leptin_ and BMI (β, 0.831, 0.486, 0.384, 0.311, 0.347, 0.216 and 0.384, respectively) was significantly higher than the association between the individual SNP,GPS_leptin_ and BMI adjusted for leptin (β Δ 0.413, 0.298, 0.214, 0.225, 0.296, 0.168 and 0.245, respectively), indicating that leptin mediated part of the association. The Sobel test confirmed that leptin significantly mediated the association between the SNPs and BMI (*P* = 0.002, *P* = 0.003, *P* = 0.007, *P* = 0.008, *P* = 0.003, *P* = 0.027 and *P* < 0.001, respectively). All the linear regression coefficients (β) were adjusted for gender, age, Tanner stage and residence.

First, we tested whether the associations between SNPs/gene score and leptin levels were independent of BMI. As shown in Table 4, these significant relationships were ablated after further adjustment for BMI, indicating that the associations of these obesity-related loci with leptin were likely not due to gene pleiotropy. Next, since all the genes at these leptin-increasing loci were found to be expressed in the CNS (Table 4), we tested if leptin plays a mediation role in genetic predisposition to polygenic obesity. Thus, leptin levels were added in to a multiple regression model to predict BMI from each of the selected six leptin-related SNPs plus their addictive score i.e. GPS_leptin_. As listed in Figure 1, the relationships were attenuated between *FTO, MC4R, GNPDA2* and BMI (β from a model without leptin: 0.831, 0.486, and 0.384, respectively, see Table 2, and β from a model including leptin: 0.418, 0.188, and 0.170, respectively and βΔ 0.413, 0.298, and 0.214, respectively), and the relationships were lost between *PCSK1, MAP2K5, BDNF* and BMI (β from a model without leptin: 0.311, 0.347 and 0.216, respectively, β from a model including leptin: 0.086, 0.051 and 0.048, respectively, and β Δ 0.225, 0.296 and 0.168, respectively). Moreover, the Sobel test confirmed significant mediation of the association between this genetic risk and BMI by leptin levels (all *P* < 0.05). As expected, we also found stronger mediation effect of leptin on the relationship between gene scores consisting of all six leptin-related loci (GPS_leptin_) and BMI than any single SNP (*P* < 0.001) (Figure 1). The mediation effect of incremental leptin on the GPS_leptin_-BMI association was estimated at 63.8%, with a significant total indirect effect of 0.245.

### Functional interactions between leptin, leptin receptor and genes associated with leptin and obesity

**Figure 2:**
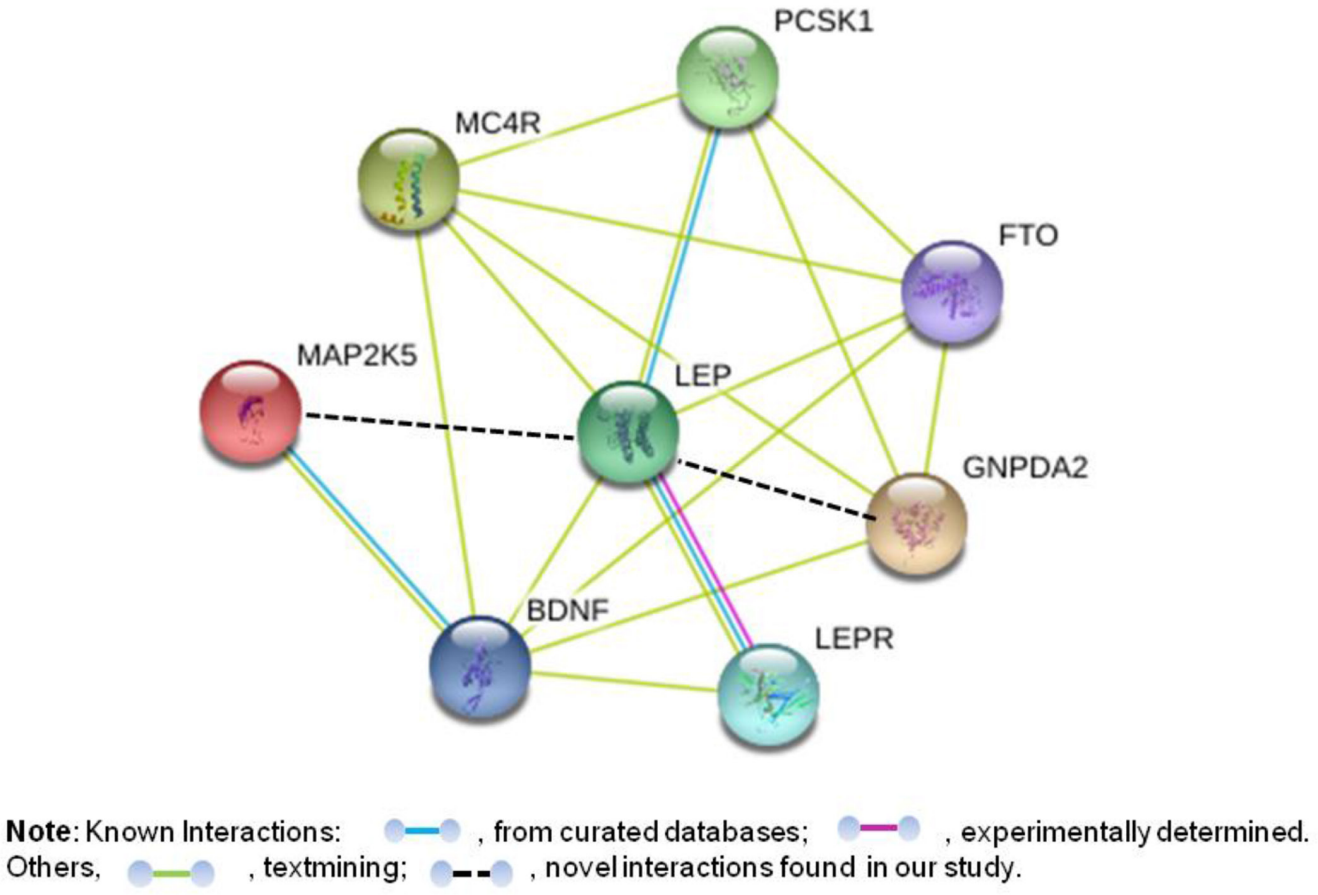
Possible interaction between the genes with at the six leptin-associated loci plus LEP and LEPR This is a prediction of the interaction between the key proteins, including *PCSK1*, proprotein convertase subtilisin/kexin type 1; MC4R, melanocortin 4 receptor; *FTO*, fat mass and obesity associated; *MAP2K5*, mitogen-activated protein kinase 5; *GNPDA2*, glucosamine-6-phosphate deaminase 2; *BDNF*, brain-derived neurotrophic factor; *LEP*, leptin and *LEPR*, leptin receptor. The prediction was conducted by using a STRING interaction network.

To further explore the interactions of the six leptin-increasing loci and *LEP* or *LEPR*, the functional interactions among these genes were predicted via the STRING database v10. As listed in Figure 2, the enrichment indicates that these genes as a group are at least partially biologically connected (clustering coefficient: 0.783). In addition, as expected, four of the six genes at these leptin-increasing loci (*PCSK1, MC4R, FTO* and *BDNF*) have interactions with *LEP*, while *BDNF* has an interaction with *LEPR*. Unlike those loci are parts or have well-established connections with the leptin pathway in literature, the interactions between the two loci in/near *MAP2K5* and *GNPDA2* and leptin were firstly found in our research.

### Associations of obesity predisposing genes with cardio-metabolic traits

For other cardiometabolic traits (Table 5), the obesity predisposing locus, *FTO*-rs1558902 yielded significant association with systolic blood pressure (SBP) (*P* = 0.001)*,* while the risk alleles of SNPs in/near *GNPDA2*, *PCSK1* and *MAP2K5* yielded nominal association with blood pressures (*P* < 0.05). In addition, the risk alleles of SNPs in/near *GNPDA2, MAP2K5*, *PCSK1* and *MC4R* revealed association with serum lipid levels (0.006 ≤ *P* ≤ 0.049). Moreover, the risk allele of *MC4R-*rs2331841 showed significant association with glucose, insulin and HOMA-IR levels (0.001 ≤ *P* ≤ 0.004)*,* while the risk alleles of SNPs in/near *GNPDA2, PCSK1, FTO* and *ITIH4* were nominally associated with glucose/insulin traits (*P* < 0.05). Meanwhile, both GPS_all_ and GPS_leptin_ yield significant association with blood pressures, HDL-C, insulin and HOMA-IR at Bonferroni- corrected levels.

When further adjusted for BMI (Table 6), the obesity predisposing loci, *GNPDA2*-rs16858082 (*P* = 0.016) and *PCSK1*-rs261967 (*P* = 0.026) were still nominally associated with total cholesterol (TC) levels, and *MC4R*-rs2331841 remained correlated with fasting glucose (FBG) levels (*P* = 0.012). Interestingly, the adult obesity-related locus *KCNQ1*-rs2237892 which was not replicated the association with childhood obesity in our study yield significant association with HDL-C (*P* = 3.7×10^−4^) levels independent of BMI.

**Table 6:** Associations of the 12 SNPs and GPSs with obesity-related and other cardio-metabolic traits after adjusting for BMI

Gene	SNP		WC (cm)	Percent body fat	UAC (cm)	SBP (mmHg)	DBP (mmHg)	TC (mmol/L)	TG (mmol/L)^#^	LDL-C (mmol/L)	HDL-C (mmol/L)	FBG (mmol/L)	Insulin (mU/L)^#^	HOMA-IR^#^
*FTO*	rs1558902	*β*	-0.084	-0.053	0.138	0.301	0.257	-0.023	-0.022	-0.024	0.012	-0.022	-0.019	-0.022
		*P*	0.561	0.746	0.017	0.451	0.419	0.444	0.165	0.380	0.241	0.273	0.407	0.359
*MC4R*	rs2331841	*β*	0.052	0.155	0.061	-0.096	-0.121	-0.009	0.010	-0.012	-0.002	0.040	0.021	0.028
		*P*	0.640	0.220	0.171	0.754	0.620	0.684	0.423	0.572	0.852	0.012	0.222	0.123
*GNPDA2*	rs16858082	*β*	0.036	0.038	-0.066	0.306	0.103	-0.048	-0.008	-0.037	-0.010	0.012	0.023	0.025
		*P*	0.712	0.735	0.091	0.258	0.634	0.016	0.434	0.046	0.144	0.396	0.128	0.115
*SEC16B*	rs516636	*β*	0.081	-0.059	-0.043	0.067	-0.154	0.002	0.003	0.001	-0.002	0.005	0.007	0.009
		*P*	0.490	0.656	0.360	0.833	0.547	0.932	0.833	0.981	0.789	0.749	0.696	0.645
*PCSK1*	rs261967	*β*	0.049	-0.135	0.018	-0.040	0.245	-0.044	-0.001	-0.031	-0.006	-0.018	0.013	0.012
		*P*	0.609	0.221	0.635	0.879	0.247	0.026	0.896	0.089	0.365	0.177	0.369	0.452
*MAP2K5*	rs4776970	*β*	0.155	0.175	0.057	0.247	0.153	-4.0E-4	0.019	-0.006	-0.013	-0.020	0.005	0.001
		*P*	0.158	0.162	0.196	0.415	0.525	0.986	0.107	0.765	0.102	0.194	0.759	0.956
*ITIH4*	rs2535633	*β*	0.028	0.098	-0.026	-0.058	-0.083	0.032	0.012	0.034	-1.7E-4	-0.004	0.015	0.014
		*P*	0.771	0.373	0.496	0.826	0.693	0.101	0.267	0.059	0.980	0.777	0.306	0.371
*BDNF*	rs2030323	*β*	-0.105	-0.04	0.007	-0.587	-0.434	0.016	-0.003	0.013	0.003	-0.008	0.009	0.007
		*P*	0.272	0.713	0.850	0.026	0.039	0.417	0.800	0.455	0.662	0.578	0.527	0.641
*ADCY3/RBJ*	rs6545814	*β*	0.121	0.07	-0.012	0.060	0.202	-0.007	-0.006	0.004	-0.002	-0.022	0.006	0.002
		*P*	0.211	0.528	0.762	0.822	0.341	0.707	0.568	0.815	0.811	0.109	0.704	0.876
*KCNQ1*	rs2237892	*β*	0.114	0.028	0.003	-0.295	0.002	-0.042	0.018	-0.028	-0.026	0.015	0.003	0.006
		*P*	0.262	0.812	0.937	0.291	0.994	0.044	0.102	0.142	**3.7E-4**	0.291	0.852	0.735
*PAX6*	rs652722	*β*	0.106	0.077	-0.028	0.283	0.155	-0.015	-0.010	-0.008	-0.007	-0.016	0.001	-0.002
		*P*	0.290	0.504	0.481	0.304	0.481	0.452	0.365	0.680	0.323	0.271	0.948	0.916
*GP2*	rs12597579	*β*	0.119	-0.108	-0.003	-0.045	0.047	0.015	0.005	0.005	0.004	-2.8E-4	-0.017	-0.017
		*P*	0.264	0.375	0.948	0.878	0.839	0.492	0.645	0.794	0.619	0.985	0.315	0.330
*GPS*_*all*_	all 12 loci	*β*	0.057	0.023	0.001	0.033	0.04	-0.01	0.002	-0.005	-0.005	-0.002	0.009	0.009
		*P*	0.065	0.512	0.939	0.702	0.562	0.139	0.540	0.389	0.019	0.634	0.050	0.072
*GPS*_*leptin*_	six leptin- related loci	*β*	0.017	0.018	0.022	-0.024	-0.006	-0.019	1.7E-4	-0.015	-0.004	-0.001	0.012	0.012
		*P*	0.696	0.723	0.204	0.843	0.952	0.036	0.972	0.064	0.242	0.826	0.081	0.099

β means linear regression coefficients adjusted for gender, age, Tanner stage, residence and BMI.

Abbreviation: BMI, body mass index; WC, waist circumference; UAC, upper arm circumference; SBP, systolic blood pressure; DBP, diastolic blood pressure; TC, total cholesterol; TG, triglycerides; LDL-C, low-density lipoprotein cholesterol; HDL-C, high-density lipoprotein cholesterol; FBG, fasting blood glucose; HOMA-IR, homeostasis model assessment for insulin resistance; *FTO*, fat mass and obesity associated; *MC4R*, melanocortin 4 receptor; *GNPDA2*, glucosamine-6-phosphate deaminase 2; *SEC16B,* SEC16 homolog B, endoplasmic reticulum export factor; *PCSK1*, proprotein convertase subtilisin/kexin type 1; *MAP2K5*, mitogen-activated protein kinase 5; *ITIH4,* inter-alpha-trypsin inhibitor heavy chain family, member 4; *BDNF*, brain-derived neurotrophic factor; *ADCY3/RBJ*, adenylate cyclase 3; *KCNQ1*, potassium channel, voltage gated KQT-like subfamily Q, member 1; *PAX6*, paired box 6; *GP2,* glycoprotein 2; GPS_all_, sum gene score of all 12 loci and GPS_leptin_, sum gene score of six leptin-related loci.

^#^ Skewed distributions were natural logarithmically (ln) transformed.

Note: Values in bold are significant at *P* < 0.004 after Bonferroni correction.

## DISCUSSION

This is the first study to systematically investigate the associations of twelve obesity/BMI-related loci, which had been previously shown to be evident in East Asian adults [12-14], with adipokine profiles and risk factors for obesity, hypertension, dyslipidemia, and diabetes in a large cohort of Chinese school-aged children. We successfully replicated the association with childhood obesity for eight SNPs in/near *FTO*, *MC4R*, *GNPDA2*, *PCSK1*, *SEC16B*, *MAP2K5, ITIH4* and *BDNF,* with odds ratio ranging from 1.13 to 1.42, implicating the transferability of susceptibility between Chinese and Europeans both in adulthood and childhood [12-14]. Notably, of these associated loci, six SNPs yielded significant or nominal association with leptin levels except for *SEC16B* and *ITIH4*. Moreover, the cumulated genetic scores i.e. GPS_all_ and GPS_leptin_ showed much stronger association with increasing leptin levels and metabolic traits. The overall variation in adiposity explained by the 6 leptin related loci and 12 BMI related loci were 1.5% and 1.4%, which is an improvement over the previously reported value of ∼1.2% in East Asian adults including 22 BMI-associated loci [12]. For cardio-metabolic risk factors, the risk allele of *KCNQ1*-rs2237892 yield significant association with decreasing HDL-C levels and *MC4R*-rs2331841 showed association with increasing FBG levels independent of BMI. These cross-phenotype associations may either reflect pleiotropy, where a gene product influences multiple metabolic traits, and/or mediation effects, where one phenotype is causally related to a second phenotype [21].

One of the key novel findings of this study is the association of the BMI-increasing loci with increasing leptin levels. Leptin is a peptide hormone produced by adipose tissue which plays a role in regulating appetite, thermogenesis, lipid oxidation and insulin sensitivity, with each of these effects attenuated in obese individuals with leptin resistance, particularly in the CNS [19]. The associations between BMI-increasing loci and increasing leptin levels may partly explain the definitive identification of the specific mechanisms about these loci influence BMI and obesity. Firstly, six of these loci (in/near *PCSK1*, *MC4R*, *FTO*, *MAP2K5*, *GNPDA2* and *BDNF*) showed a directionally concordant association with increasing leptin levels as well as increasing adiposity. Second, the predicted functional interactions among these genes and *LEP/LEPR* indicate that these genes as a group are at least partially biologically connected. In addition, mediation analysis revealed that leptin levels significantly mediated the association observed for genetic predisposition to adiposity. It should be noted that all the six leptin-increasing loci highlighted genes are expressed in and/or known to act in the brain (and several particularly so in the hypothalamus) [17, 22-24]. Therefore, these findings suggest that as is seen in monogenic forms of obesity, inherited variations in these leptin-increasing loci influence common obesity may through their effects in the CNS, particularly pointing to neuronal “leptin resistance”, thus, highlighting a possible neuronal influence on body weight regulation in children comparable to previous reports in adults [17].

In addition to leptin, we also found nominal association between adiponectin levels and three hypothalamic leptin–melanocortin pathway-related genes (*MC4R, BDNF* and *PCSK1*). Adiponectin is an insulin-sensitizer expressed in adipose tissue. A decrease in the circulating levels in obesity has been shown to contribute to the development of insulin resistance, T2D and metabolic syndrome [25]. Besides its peripheral actions, adiponectin has also been demonstrated to play an important role in the CNS: being present in the cerebral spinal fluid and enhancing AMP-activated protein kinase activity in the arcuate hypothalamus to stimulate food intake and decreases energy expenditure [26]. Thus, our findings suggest that the pathophysiology of obesity signaling in the CNS may not only influencing adiposity but also play an important role in the regulation of adiponectin levels.

We did not find any significant relationship between these BMI-increasing loci and other three important adipokines: resistin [19], FGF21 [19] and RBP4 [20], which can also function via the CNS. However, unlike adipokine leptin and adiponectin which are mainly produced by adipocytes, resistin is mainly secreted by mononuclear cells in humans, promoting both inflammation and IR [27]; FGF21 is primarily produced by liver and is associated with beneficial metabolic characteristics, including reduced body weight and hepatic steatosis, as well as improved insulin sensitivity and lipid/glucose profiles [28]; while RBP4 is secreted primarily by liver and adipocytes, playing an important role in the development of obesity and other metabolic abnormalities by inducing adipose tissue inflammation and promoted systemic IR [29]. Thus, the differing source of production may provide an explanation for the lack of association of the three adipokines with the BMI-related loci.

Among the six leptin-associated loci, three genes (*MC4R, BDNF,* and *PCSK1*) are known to be involved in the hypothalamic leptin–melanocortin pathway [9, 17, 30], thus it is not surprising that those three loci are found to be associated with obesity-related traits and leptin levels in our pediatric setting. Indeed, our findings suggest a potential effect of *MC4R* on glucose homeostasis beyond obesity. Given the fact that *MC4R* encodes the melanocortin-4 receptor in the leptin-melanocortin signaling pathway [17, 30], wherein leptin up-regulates anorexigenic neuropeptides such as alpha-melanocyte-stimulating hormone, which acts on the melanocortin-4 receptor to control energy intake and metabolic regulation, our findings, therefore, implicate that the role of *MC4R* in controlling glucose metabolism may also depend on leptin pathways.

*FTO* is the first GWAS-identified obesity-susceptibility locus; indeed, like many of the other replication efforts, *FTO* shows the strongest association with BMI in our pediatric cohort and accounts for the largest proportion of the variance (0.5%), which is higher than its estimated by East Asian adults (∼0.15%) [14]. However, unlike the above-noted three genes with possible function through hypothalamic leptin–melanocortin pathway, the process by *FTO* impacts on obesity pathogenesis has remained elusive [17]. Given that available experimental evidence has shown that *FTO* modulates leptin receptor localization within neurons to control food intake and adiposity [31], our finding provides an additional insight into the linkage between *FTO,* leptin and adiposity, and obesity-related characters.

Notably, among the six leptin-associated loci, the two in/near *MAP2K5* and *GNPDA2*, have not been reported before in this context. In consideration of the limited knowledge of the associations, particularly in the early years, our results highlight the need to further investigate the complex relationship between leptin and *MAP2K5* and *GNPDA2.*

When compared the accumulated genetic effects by above six leptin-associated loci (GPS_leptin_ ) with all twelve selected loci (GPS_all_), GPS_leptin_ showed equal or even more significant correlations with adiposity-traits than GPS_all_, thus, the six leptin-increasing SNPs may be used as a novel set of biomarkers to identify the risk of obesity. Since all the leptin-related loci were expressed and/or functioned in CNS, therefore, suggesting a possible central role of the six loci in the process of body weight regulation, and providing a potential therapeutic targets to prevent obesity.

Besides the six leptin-increasing SNPs, we also replicated the associations of other three loci in *SEC16B, ITIH4* and *ADCY3/RBJ* with adiposity traits, but no relationship between the three loci and leptin levels was observed, suggesting the possible different mechanisms between these loci underlying the body weight regulation, and the specific mechanisms through which these loci affect BMI and obesity require further study.

In addition, despite our study being sufficiently powered, we failed to confirm the association with obesity for three novel identified adult BMI loci from East ancestry (*KCNQ1*-rs2237892, *PAX6*-rs652722 and *GP2*-rs12597579) in Chinese children [12, 14]. The reason for the results deviating from what is seen in adults may owe to the genes operating differently in childhood compared to adulthood [9]. Thus, separating genetic component that is exclusively associated with childhood obesity remains to be explored.

However, we found a strong connection between the risk allele of *KCNQ1*-rs2237892 and blood lipids, especially with the HDL-C levels. In line with our results, a study in a middle-aged Chinese Han population also found subjects with CC genotype in another locus rs2283228 in *KCNQ1* (r^2^_CEU_ = 0.867 with rs2237892) had lower levels of HDL-C [32]. Given that patients with obesity and T2D are more likely to have lower levels of HDL-C and large prospective studies have identified HDL-C as a strong, independent, inverse predictor of risk of CVD [33], the strong association of *KCNQ1* variant with HDL-C in our pediatric population may provide a novel insight of the link between *KCNQ1* and early risk in the pathogenesis of T2D and CVD.

## MATERIALS AND METHODS

### Population

Subjects were recruited via a cross-sectional population based survey: the Beijing Child and Adolescent Metabolic Syndrome (BCAMS) study [27]. This study evaluated the prevalence of obesity and related metabolic abnormalities (hypertension, hyperglycemia and dyslipidemia) among a representative sample of Beijing school-aged children and adolescents (n = 19593, ages 6 to18 years, 50% male) between April and October 2004. Within this cohort, 4500 subjects were identified as having one or more of the following disorders: Being overweight defined by BMI percentile, increased TC ≥ 5.2 (mmol/L), TG ≥ 1.7 (mmol/L) or FBG ≥ 5.6 (mmol/L) based on finger capillary blood tests. Age- and sex-specific BMI percentiles, according to the Working Group for Obesity in China, were used to define overweight (85^th^) and obesity (95^th^) [34]. Totally 3,506 subjects, including 1,024 normal controls, agreed to complete further medical examination, thus were included for this study. The BCAMS study was approved by the ethics committee of the Capital Institute of Pediatrics and all the methods were performed in accordance with relevant guidelines and regulations. Signed informed consents were obtained from all participants and/or their parents or guardians through all the study processes.

### Phenotyping

Subjects’ height, waist circumference (WC), weight, percent body fat, upper arm circumference (UAC), systolic blood pressure and diastolic blood pressure (SBP and DBP) were measured by trained recruiters with standard methods. Height was measured to the nearest 0.1 cm using a portable stadiometer. WC was measured midway between the lowest rib and the top of the iliac crest. Weight and percent body fat was measured to the nearest 0.1 kg using a TANITA Body Composition Analyzer (Model TBF–300A). UAC was measured at the mid-point between the tip of acromion process and olecranon process of the right upper arm. Measurements of right arm SBP and DBP were performed 3 times at 10 minutes apart and the mean values of the latter two measurements were recorded. BMI was calculated as weight divided by height squared.

Venous blood samples were collected after an overnight (≥ 10 h) fast. The samples were centrifuged, and immediately frozen for future analysis of hormones. Serum lipids (enzymatic methods) and FBG (glucose oxidase method) were assayed using the Hitachi 7060 C automatic biochemistry analysis system. Insulin, leptin and adiponectin were measured by sandwich enzyme-linked immunosorbent assay (ELISA) developed in the Key Laboratory of Endocrinology, Peking Union Medical College Hospital. The insulin assay had an inter-assay coefficient of variation (CV) of < 9.0% and no cross-reactivity to proinsulin (< 0.05%) [34]. Insulin resistance index was calculated by homeostasis model assessment as [HOMA-IR= (fasting insulin IU/L) ×; (FBG mmol/L)/22.5].The intra-assay and inter-assay CVs for leptin were < 7.4% and < 9.3%, respectively and < 5.4% and < 8.5% for adiponectin, respectively [35, 36]. Serum resistin was measured using the ELISA kit by Phoenix Pharmaceuticals Inc. The intra-assay and inter-assay CVs of this assay were < 5.2 and < 10.1%, respectively [27]. RBP4 was measured by commercial ELISA kits (Dou set, R&D Systems, Minneapolis, MN, USA) with intra- and inter-assay CVs of 6.2% and 8.5%, respectively. FGF21 was measured by an ELISA kit (Phoenix Pharmaceuticals, Burlingame, CA, USA) with intra- and inter-assay CVs of < 6.0% and < 8.6%, respectively [37]. All samples were tested in duplicate and blinded.

### SNP selection and genotyping

Thirteen SNPs were selected from GWAS reports of adult obesity in East Asian ancestry populations [12-14]. Among them, eight SNPs: *FTO*-rs1558902, *MC4R*-rs2331841, *BDNF*-rs2030323, *ADCY3/RBJ*-rs6545814, *GIPR/QPCTL*-rs11671664, *MAP2K5*-rs4776970, *GNPDA2*-rs16858082, and *SEC16B*-rs516636 were initially identified in European-ancestry populations which went on to satisfy the genome-wide significance threshold in East Asian-ancestry subjects [12-14], while the five other loci, *KCNQ1*-rs2237892, *PCSK1*-rs261967, *PAX6*-rs652722, *GP2*-rs12597579, and *ITIH4*-rs2535633 were first identified in adult BMI studies in East Asians [12, 14].

Genomic DNA was isolated from peripheral white blood cells using the QIAamp DNA Blood Midi Kits (Qiagen). All SNPs were genotyped on the Sequenom Mass Array iPLEX genotyping platform in BioMiao Biological Technology (Beijing) Co, Ltd. [38] Repeated control samples were present in each genotyping plate with the concordance rate being 100%. SNPs were excluded if they had genotyping efficiency less than 0.95, or a Hardy-Weinberg equilibrium *P* < 0.0042 (0.05/12). Except for *GIPR/QPCTL*-rs11671664, twelve SNPs were in Hardy-Weinberg equilibrium, thus incorporated in the present study.

### Statistical analysis

Analyses were performed using Statistical Package for Social Sciences (SPSS) 19.0 for Windows. Hardy-Weinberg equilibrium was assessed using the chi-squared test in controls. By applying Bonferroni correction, a *P*-value below 0.0042 (0.05 divided by 12 SNPs) was considered significant, while a *P*-value between 0.05 and 0.0042 was considered nominally significant. All skewed distributions were naturally logarithmically transformed for analysis. Continuous data are presented as mean ± SD or geometric mean (95% CI). During the initial step, one-way ANOVA was utilized for continuous variables in relation to obesity. The adjustment for confounding factors was performed using the analysis of covariance in the general linear model (GLM). A score of 0, 1 or 2 was assigned to genotypes of associated SNPs according to the number of risk alleles in the additive model. To evaluate the combined effect of the SNPs on obesity and metabolic phenotypes, we generated two different genetic predisposition scores (GPSs) (GPS_all_: consisting of all twelve SNPs; GPS_leptin_: all leptin-related SNPs) by summing the number of risk alleles that each subject carried at each SNP. Logistic or linear regression analysis was then applied to examine the associations between each SNP or category of GPS tertile and obesity risk, or related phenotype, with adjustment for confounders. We obtained the pairwise distance and LD (r^2^) data from the International HapMap Project and derived the predicted associations among proteins using the publically available STRING database (http://string-db.org). The post-hoc power of the study was estimated using G^*^power software program [39]. The sample sizes revealed > 99% power to detect a significant association (α < 0.05), given an effect size index of 0.1 (corresponds to a “weak” gene effect). The Sobel test [40] was used to assess whether leptin significantly mediated the association between the leptin-related SNPs, gene score and BMI.

## CONCLUSIONS

In summary, in our sample of Chinese children, we replicated eight adult East Asian obesity-related loci (in/near *FTO*, *MC4R*, *GNPDA2*, *PCSK1*, *SEC16B*, *MAP2K5*, *ITIH4* and *BDNF*) in the same direction of effect as previous reports in adults. Moreover, the association of the harboring brain-expressed loci with leptin highlights a neuronal-related influence on body weight regulation in children. Interestingly, independent of BMI, *MC4R*-rs2331841 yielded an association with increased FBG levels and *KCNQ1*-rs2237892 with decreased HDL-C levels, indicating that the risk alleles at *MC4R* and *KCNQ1* play a particular role in the regulation of glucose and lipid profiles besides their function in body weight control. The precise mechanism by which these loci act on BMI remains to be determined; as such, further studies are warranted to support this supposition and to give us more evidence.

## Abbreviations

BMI: body mass index
WC: waist circumference
UAC: upper arm circumference
SBP: systolic blood pressure
DBP: diastolic blood pressure
TC: total cholesterol
TG: triglycerides
LDL-C: low-density lipoprotein cholesterol
HDL-C: high-density lipoprotein cholesterol
FBG: fasting blood glucose
HOMA-IR: homeostasis model assessment for insulin resistance
FGF21: fibroblast growth factor 21
RBP4: retinol binding protein 4
GPS_all_: sum gene score of all 12 loci
GPS_leptin_: sum gene score of six leptin-related loci
